# Supplementary material for: The Risk Factors Associated with the Carriage to Critical Antimicrobial-Resistant Escherichia coli in Healthy Household Dogs: A One Health Perspective
Source: Animals (Basel). 2025 May 8;15(10):1357. doi: 10.3390/ani15101357 (PMC12108178; doi:10.3390/ani15101357)
Supplement: Supplementary file 1 [file animals-15-01357-s001.zip › animals-3597185-supplementary.pdf]

Supplementary data:

Table S1: Summary of answers extracted from the owner's questionnaires (n = 263).

	Responses		Reyesstant Dogs		
Variable	Value	N	AMC	CTZ	ENR
Place where the pet sleeps	Inside the house	208	27	13	37
	Outside the house	65	9	2	11
	Undetermined	28	4	3	7
Origin of the pet	Adoption	227	31	8	34
	Purchase	74	9	10	21
Pet's gender	Female	158	21	6	24
	Male	143	19	12	31
Proximity of the home to a healthcare establishment (veterinary or human)	No	55	5	1	10
	Yes	246	35	17	45
Age	Between 2 and 10 years old	174	22	8	31
	Older than 10 years	51	8	0	7

	Younger than 2 years	76	10	10	17
Owner's age	Older than 50 years	44	7	3	9
	Younger than 50 years	257	33	15	46
Place where the pet mainly reyesdes	Inside the house/apartment	106	17	8	22
	Outside the house/apartment	74	9	5	12
	Both outside and inside the house	59	0	0	0
	Both outside and inside the house/apartment	62	14	5	21
Level of contact between household members and the pet	High (kisses and hugs)	260	36	16	46
	Low (only during feeding and/or taking for walks)	33	4	2	9
	Undetermined	8	0	0	0
Breed of the pet	Mixed breed	176	27	10	31
	Purebred	125	13	8	24
If the pet lives with other animals, have these other animals	No	266	33	18	49
	Yes	35	7	0	6

been hospitalized in the past year?					
If you purchase dry processed food, in what format do you buy it?	In bulk	33	6	3	9
	Sealed package	268	34	15	46
yesze of the pet	Large (over 27kg)	36	5	1	6
	Medium (between 11-27kg)	112	16	7	15
	Small (under 11kg)	153	19	10	34
Type of houyesng of the owner	House	226	30	11	38
	Apartment	75	10	7	17
Has any family member been hospitalized or required antibiotic treatments during 2020–2021?	No	202	28	17	37
	Yes	99	12	1	18
	Homemade food	4	3	0	0
	Commercial food	266	32	17	49

What is the primary type of food or diet your pet receives?	Commercial and homemade food	31	5	1	6
When you walk your pet, do you use a leash?	No	59	16	4	14
	Yes	242	24	14	41
Where do you buy this food?	Large-scale commercial establishment (supermarkets, pet shop)	254	35	15	41
	Small-scale commercial establishment (corner store, market)	24	4	1	6
	Both large- and small-scale establishments	23	1	2	8
Has your pet been dewormed in the last 6 months?	No	45	10	4	7
	Yes	256	30	14	48
Have you observed your pet drinking water from springs or	No	248	31	17	40
	Yes	53	9	1	15

stagnant sources in the environment?					
Have you observed your pet ingesting garbage and/or food scraps?	No	170	31	17	40
	Yes	131	14	6	29
Have you observed your pet ingesting feces (its own or others')?	No	213	27	12	36
	Yes	88	13	6	19
Has your pet received antibiotic treatments during its lifetime?	No	117	12	7	19
	Yes	184	28	11	36
Has it been necessary to hospitalize your pet during its lifetime?	No	217	27	11	32
	Yes	84	13	7	23
Has your pet been vaccinated in the past year?	No	59	11	2	9
	Yes	242	29	16	46
Is the water source for your pet drinkable?	No	9	0	0	2
	Yes	292	40	18	53

Do you add raw meat/bones to your pet's diet?	No	244	38	17	49
	Yes	57	2	1	6
Do you give treats to your pet?	No	63	11	2	7
	Yes	238	29	16	48
Have you administered antibiotics to your pet without consulting a veterinarian?	No	296	39	18	54
	Yes	5	1	0	1
Do you pick up your pet's feces during walks?	No	50	9	3	8
	Yes	251	31	15	47
Does your pet live with or have contact with other animals that are under your care or responyesbility?	No	83	9	10	21
	Yes	218	31	8	34
Is your pet sterilized or neutered?	No	94	10	5	22
	Yes	207	30	13	33
	No	61	5	0	7

Does your pet go out for walks during the week?	Yes	240	35	18	48
Does your pet go out alone or escape from the home?	No	241	6	2	11
	Yes	60	0	0	0
Does your pet have any chronic illnesses?	No	258	37	17	47
	Yes	43	3	1	8
Does your pet have contact with animals that are NOT under your care or responyesbility?	No	154	27	7	24
	Yes	147	13	11	31
Do you or any member of your family who lives with your pet work in a human or veterinary healthcare establishment (such as a hospital, clinic, dentist, production systems, or	No	190	30	12	43
	Yes	111	10	6	12

recreational/producti<br><br>on animal farms)?					

Table S2: Univariable logistic regression AMC.

Factor	p-value
X1	0,7537
X2	1
X3	0,9208
X4	0,4267
X5	0,8125
X6	0
X7	0,971
X8	0,513
X9	0,3973
X10	1
X11	0,8528
X12	0,901
X13	0,2837
X14	0,8952
X15	0,5218
X16	0,4655
X17	0,6127
X18	0,2883
X19	0,4729
X20	1
X21	0,2553
X22	0,0937
X23	0,2826
X24	0,5808
X25	0,001
X26	0,3894
X27	0,5447
X28	0,0224
X29	0,4877
X30	0,5161
X31	0,3192
X32	0,7636
X33	0,3745
X34	0,561

X35	0,0404
X36	0,3274
X37	0,2709
X38	0,0011
X39	0,5312

Table S3: Univariable logistic regression CTZ.

Factor	p-value
X1	1
X2	0,2575
X3	0,012
X4	0,2604
X5	0,0222
X6	0,0078
X7	0,3469
X8	0,7698
X9	1
X10	0,1512
X11	0,0045
X12	0,6855
X13	0,9902
X14	0,0042
X15	0,0179
X16	0,9493
X17	0,4235
X18	1
X19	0,927
X20	1
X21	0,5289
X22	0,5813
X23	0,4567
X24	0,528
X25	0,6864
X26	0,8007
X27	0,682
X28	0,2143
X29	0,9565
X30	0,2867
X31	0,5131

X32	0,899
X33	0,4488
X34	0,0136
X35	0,4058
X36	0,227
X37	0,057
X38	1
X39	0,5079

Table S4: Univariable logistic regression ENR.

Factor	p-value
X1	0,846
X2	0,335
X3	0,322
X4	1
X5	1
X6	0
X7	0,6186
X8	0,1622
X9	0,7987
X10	0,1918
X11	0,4529
X12	0,1785
X13	0,8418
X14	0,0156
X15	0,0026
X16	0,164
X17	0,0174
X18	0,5654
X19	0,2936
X20	1
X21	0,6304
X22	0,7625
X23	1
X24	0,6994
X25	0,6305
X26	0,0579
X27	0,2383
X28	0,1078
X29	1

X30	0,0593
X31	0,1698
X32	0,4274
X33	0,1413
X34	0,075
X35	0,2775
X36	1
X37	0,1761
X38	0,3069
X39	1

Table S5: Goodness-of-fit and model significance statistics for the multivariate logistic regression models evaluating resistance to amoxicillin-clavulanic acid (AMC), ceftazidime (CTZ), and enrofloxacin (ENR) in E. coli isolates from dogs.

Model	Null Deviance	Residual Deviance	Degrees of Freedom (df)	Deviance/df Ratio	LRT (Null - Residual)	LRT Degrees of Freedom
Amoxicillin/Clavulanic acid (AMC)	235,89	166,1	293	0,57	69,79	7
Ceftazidime (CTZ)	136,3	88,04	292	0,3	48,26	8
Enrofloxacin (ENR)	286,25	187,13	290	0,65	99,12	10

Questionnaire:

SECTION S1: GENERAL INFORMATION OF THE CAREGIVERS

1.- Pet Owner's Name

2.- Dog Identification (Name and Tag Number)

3.- Age:
18-30 years
Between 30-50 years
Over 50 years

4.- Family Home Address

5.- Type of Caregiver’s Residence:
House with yard
House without yard
Apartment with balcony
Apartment without balcony

6.- Indicate the number of people by category living with the animal, based on the following ranges: Babies (under 1 year); Children and adolescents (1 to 18 years); Adults (between 18-60 years); Seniors (over 60 years)

7.- Is your home located near a human or veterinary health facility?
Yes
No

8.- Has any member of your family been hospitalized or required antibiotic treatments in the past year?
Yes
No

9.- Do you or any member of your family living with your pet work in a human or veterinary health facility?

10.- Which of the following activities do you do with your pet?

11.- Do you pick up your pet's feces when going for a walk or at home?

SECTION S2: GENERAL INFORMATION ABOUT THE PET

12.- Sex:
Female
Male

13.- Age:
Under 2 years
Between 2 and 10 years
Over 10 years

14.- Pet Size:
Small (under 11kg)
Medium (between 11-27kg)
Large (over 27kg)

15.- Pet Breed:
Purebred
Mixed breed

16.- If your previous answer was "purebred," what is your pet's breed?

17.- Origin of the Pet

18.- How long have you been living with your pet?

19.- Is your pet spayed or neutered?
Yes
No

SECTION S3: CLINICAL INFORMATION OF THE DOG

20.- Has it been necessary to hospitalize your pet at any point in its life?
Yes
No

21.- Has your pet ever received antibiotic treatment in its lifetime?
Yes
No

22.- If your answer to the previous question was yes, how was the treatment administered?

23.- Have you given your pet antibiotics without consulting a veterinarian?
Yes
No

24.- Has your pet been vaccinated in the past year?
Yes
No

25.- Has your pet been dewormed in the last 6 months?
Yes
No

26.- Does your pet have any chronic illnesses?
Yes
No

27.- If your answer to the previous question was yes, what chronic illness does your pet have?

SECTION S4: ENVIRONMENT INFORMATION

28.- Primary Living Area of Your Pet

29.- What is the main type of food or diet your pet receives?

30.- If you do not prepare your pet's food yourself, where do you purchase it?

31.- If you purchase processed dry food, in what format do you buy it?

32.- Do you add any of the following supplements to your pet's diet?

33.- Is your pet's water source potable?
Yes
No

34.- Have you observed your pet consuming water from springs or stagnant sources in the environment?

Yes
No

35.- Have you observed your pet ingesting garbage and/or food scraps?
Yes
No

36.- Have you observed your pet ingesting feces (its own or others')?
Yes
No

37.- Do you give your pet treats?
Yes
No

38.- If your previous answer was yes, what type of treats do you give your pet?

39.- Does your pet live with or come into contact with other animals under your care or responsibility?

40.- Does your pet come into contact with animals that are NOT under your care or responsibility?

41.- If your pet lives with other pets, have any of these other pets been hospitalized in the last year?
Yes
No

42.- What is the weekly frequency of your pet's walks?

43.- When you go for a walk with your pet, do you use a leash?
Yes
No

44.- Does your pet go out on its own or escape from the home?
Yes
No
Sometimes

45.- If you would like to receive the results of this study's analysis, please provide your email address:
